# Learning to Drive Safely: Reasonable Expectations and Future Directions for the Learner Period

**DOI:** 10.3390/safety2040020

**Published:** 2016-10-19

**Authors:** Bruce Simons-Morton, Johnathon P. Ehsani

**Affiliations:** 1 Health Behavior Branch, Eunice Kennedy Shriver National Institute of Child Health and Human Development, Bethesda, MD 20892, USA; 2 Center for Injury Research and Policy, Department of Health Policy and Management, Johns Hopkins Bloomberg School of Public Health, Baltimore, MD 21205, USA

**Keywords:** risk taking, learning, expertise, training, translation, safety, attention, crashes

## Abstract

The young driver problem is typified by high crash rates early in licensure that decline with experience, but are higher initially and decline more slowly for the youngest novices. Despite considerable effort, only Graduated Driver Licensing System (GDLS) policies have been shown to improve novice young driver safety outcomes. Unfortunately, GDLS policies are mostly limited to countries with a relatively young licensure age. Meanwhile, it is not entirely clear how GDLS and other young driver transportation safety efforts, including driver training and testing, supervised practice and parental management of young drivers, can best be configured. Notably, professional training can foster improvements in vehicle management skills that are necessary, but do not assure safe driving behavior. Substantial recent research has focused on training methods to improve driving skills, but the safety benefits of driver training have not been established. While prolonged practice driving increases experience and provides supervisors with opportunities to prepare novices for independent driving, the transition to independent driving challenges novices to employ, on their own, poorly-mastered skills under unfamiliar and complex driving conditions. Licensing policies and parental management practices can limit the complexity of driving conditions while novices gain needed driving experience. Nevertheless, an emerging body of literature suggests that future advances in training and supervision of novice teenage drivers might best focus on the translation of learning to independent driving by fostering safe driving attitudes and norms, judgment, dedicated attention to driving tasks and self-control at the wheel.

## 1. Introduction

The high crash rate among novices during the early period of independent driving that declines with experience is known as the young driver problem. The young driver problem gives rise to the prevailing concern about how to allow novices to gain experience without increasing their crash risk. Currently available prevention programs and practices, including Graduate Driver Licensing System (GDLS) policies, supervised practice driving, driver training and parental management, have not resolved the problem.

GDLS policies have been adopted in North America, Australia and elsewhere. GDLS is an outgrowth of the realization that novices are at high crash risk when first licensed to drive independently [1,2] and, therefore, should drive for a time only under relatively less dangerous driving conditions while driving skills and judgment develop [3]. The provisions of GDLS vary by jurisdiction, but generally include a prolonged learner period, extended training and supervised practice driving requirements, and limits on independent driving during the first year or so after licensure. The extended learner period as part of GDLS policies is designed to facilitate training, increase the amount of practice and provide additional experience prior to independent licensed driving. GDLS policies in North America have generally increased the length of the learner license period from a few weeks to six months or more and the amount of required supervised practice driving to 50 h or more, not too dissimilar from the requirements in many other countries, but less than the 100 h required in Queensland and 120 h in Victoria, Australia [4,5]. Evaluations of GDLS provide evidence that adoption, particularly of more restrictive policies, reduces crash outcomes [6,7]. However, it is not clear the extent to which safety benefits of GDLS are due to delays in licensure, increases in practice driving, restrictions on driving conditions or other aspects of reduced exposure [7,8], or how best to improve GDLS effectiveness [3]. Moreover, GDLS policies have generally not been adopted by countries where licensure age is 18 or older.

With or without GDLS, the driving safety of novice drivers could be improved through more effective driver training, supervised practice driving, and parental management of independent driving. While training and supervised practice driving are integral to comprehensive approaches to preparing novices for independent driving, the extent of their contributions to safety outcomes has not been established. The efficacy of programs fostering improved parental management of independent driving has been established in small studies, but they have not proven feasible to “scale up”, extending benefits to broader populations. It is of continuing interest then to determine if and how training and supervision could improve independent driving performance and safety. The purposes of this paper are to: (1) describe how the protracted process necessary for the development of safe driving expertise limits the potential effectiveness of training and supervised practice during the learner period; and (2) suggest strategies that might improve the translation of learning from driver training, supervised practice and parent management to independent driving safety. We argue that an extended learner period coupled with improved training, supervised practice, and parental management that emphasizes the translation of learning to independent driving could improve novice driver safety.

## 2. Learning to Drive Safely

By definition, novice drivers, like novices at anything, lack experience and expertise and, therefore, make many mistakes. Indeed, making mistakes is essential to learning because mistakes provide feedback from which the driver learns. In this respect, driving is not that different from skiing, tennis, cooking, carpentry, playing a musical instrument, and other complex, psycho-motor activities. Like other complex activities, driving is a highly dynamic activity, as the road conditions change constantly, the intentions and performance of other drivers and vehicles must be judged quickly and accurately, and the driver must attend constantly to managing the vehicle while identifying and mitigating potential safety threats. Just like other complex activities, excellent performance is not possible without substantial practice and experience.

### 2.1. Expertise

To understand how novices learn to drive, it is instructive to consider the extensive literature on the development of the expertise of complex tasks. Research on the development of expertise in various areas, chess, mathematics, sport, surgery, aircraft piloting, music, art, to name a few, indicates that learning is a gradual process that occurs through extensive practice [9]. Indeed, in transportation, expertise is defined commonly by the “ … number of years operating the vehicle, miles driven, or h[ours] flown by the operator” (p. 355, [10]). Crash rates decline with experience, so that the more one drives, the lower the crash rate per mile driven [11]. Experience, then, is an important element of driving expertise.

Expertise, defined as achieving one’s highest possible skill level, requires some 10,000 h of experience, an assertion made famous by Malcolm Gladwell in the book, *Outliers* [12], based on the research of Ericsson and colleagues [9,13]. Actually, Ericsson et al. [9,13] contend that 10,000 h of deliberate practice and not just passive experience are needed. Deliberative practice is distinguished as practice with feedback and consequences. Accordingly, it is not the hours of practice, but the persistent and meaningful feedback from experience that contributes to learning. Repetition alone may be more likely to develop complacency than expertise, but when practice is dynamic, occurring in a variety of contexts, and paired with feedback and consequences, maximal improvement in performance can be achieved [13]. Of course, drivers frequently make mistakes for which they do not receive feedback in the form of a crash or other critical event. Therefore, consistent with the development of expertise in general, novice drivers need to learn about what constitutes acceptable and safe driving practices from the feedback provided by trainers and supervisors, as well as from experience itself. This sort of feedback about performance during practice improves performance, reduces errors and minimizes negative consequences. Teachers, trainers and coaches design practices so that their protégés practice correctly over and over, initially under simplified, artificial conditions, gradually varying conditions to approximate real, dynamic conditions, providing corrective feedback along the way. Of course, as every amateur coach and parent knows, even after substantial practice in well-controlled environments, when novices are initially exposed to dynamic environments, like a real soccer match or piano recital, they make unaccustomed mistakes. However, even these mistakes provide useful feedback and consequences from which learning occurs. To be sure, learning is contextual, and seeming mastery of skills under controlled conditions, while necessary, does not immediately result in correct performance under complex, real-world conditions. For that to occur, one needs extensive independent driving experience in real-world conditions.

**Figure 1:**
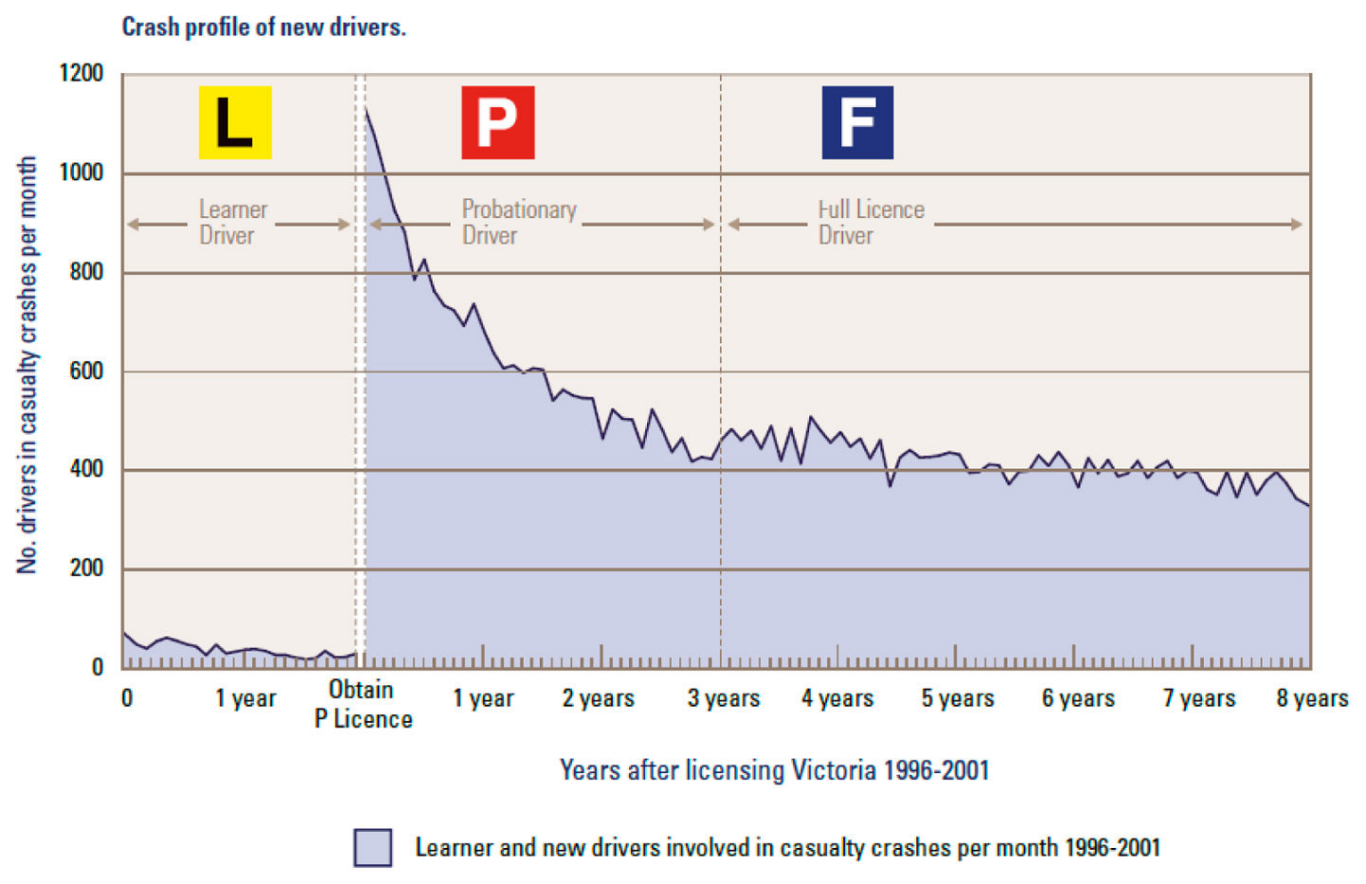
Low crash rates during the learner period, highest rates at licensure, with rapid declines for about six months, then slower declines, consistent with a classic learning curve [14].

The extent to which performance for every complex skill improves and errors decline with deliberative practice is illustrated by the classic learning curve. Think about learning to ski or ride a bicycle. At first, you could barely stay upright without direct assistance. Gradually, you learned how to navigate easy terrain and reduce falls and crashes. However, as soon as you encountered steeper slopes or bumpier ground, your risk of falling increased. Gradually with practice and many mistakes, possibly with instructional feedback, you got better and better. The learning curve for driving shown in Figure 1 reflects the highest rate of mistakes and poorest performance at the beginning, with rapid improvement, then increasingly gradual improvement as the easy skills are mastered and the more complex skills take longer, particularly because of increased exposure over time to more complex conditions with attendant challenges to skill. The figure also illustrates the dramatic increase in crash risk early in licensure relative to pre-licensed supervised practice driving, demonstrating the importance of independent, unsupervised driving practice.

Virtually every driver eventually learns to be reasonably safe, as evidenced by the low crash rates among experienced relative to novice drivers [1]. Novice drivers make many errors [11,15–17], drive in a more risky manner than experienced drivers [18,19] and fail to maintain attention to essential driving tasks [20]. The decline in crash rates among novices by months of licensure (experience), shown in Figure 1, forms a classic (inverse) learning curve, where crashes decline, presumably as safe driving behaviors improve, the greatest learning occurring during the first 1000 miles and six months of independent driving experience, then improving more gradually over a period of years. Age is a factor, with older age at licensure attenuating the learning curve, but novices at every age are at their worst and get better with experience [2].

While it is evident that risk is greatest among those with the least experience, it is not clear what novices learn with experience that improves their safety. The evidence that novices make many mistakes suggests the need for additional training, but it is not clear the extent to which training should focus on vehicle management skills, judgment relating to how and when to apply vehicle management skills or personal skills relating to attitudes, attention and self-control during independent driving. Learning to drive safely is a complicated process with multiple dimensions that are not easily measured, in part because learners accept greater challenges as their skills and confidence improve. Furthermore, it is not completely clear which skills are most important. Some argue that attention is the most important skill, while others argue that recognizing hazards is most important, while still others argue that self-control and attitudes favoring safe driving are most important. While all of these improve with driving experience, the key to resolving the young driver problem is preparing novices for the complexities of independent driving.

### 2.2. How Novices Learn

Learning theory provides insight into how novices learn (anything) and how novice drivers can best be trained during the learner period. The classic conceptualization by Fitts and Posner [21] indicates that the advance in learning complex tasks occurs in three overlapping stages described as cognitive, associative and autonomous. The cognitive stage is typified by the development of explicit knowledge, basically knowing what to do and maybe how to do it under simple conditions. Associative learning focuses on the details, sequence and application of explicit knowledge under varying and complex conditions. The autonomous stage, which can occur only after substantial practice and experience, represents the internalization of associative learning, such that learners respond effortlessly, without consciously thinking about their behavior. Sport offers abundant examples. A novice soccer player learns through practice how to maintain possession, pass and kick the ball. On the practice field over many years, the player develops these skills and improves his or her performance, but as the player gets better, so does the competition. At the highest level, advanced players have so perfected their skills and practiced so much that they can perform effortlessly, focusing on strategic issues and quickly reacting to the changing conditions on the field with little conscious effort, autonomously.

Automaticity is a well-recognized, if little studied, concept in driving. Basically, experienced drivers are thought to develop schema allowing routine, non-executive functions to control established (over-learned) skills, such that useful reactions are autonomous, without conscious effort [22]. Automaticity explains how an experienced driver can be listening to the radio, talking animatedly with a passenger or on the phone or be “lost in thought” and still react immediately to the brake lights of a lead vehicle or the subtle encroachment of an emerging vehicle. Such autonomous behavior is unlikely from novices because they lack the experience required for appropriate schema to develop. Indeed, autonomous behavior on the part of novices might be dangerous because their actions would not have been grounded in the substantial experience required for appropriate schema to develop that guide correct and safe automatic reactions.

Learning a new skill is highly demanding cognitively. Novices must marshal most of their available cognitive capacity to perform a specific activity and have no excess cognitive capacity to attend to other things. Consider juggling for example. For a novice, juggling three objects requires total concentration, and any distraction increases juggle errors. However, an expert can juggle numerous objects while eating an apple, changing clothes or engaging in other competing activities, all because juggling has been practiced to the point that it is effortless and adjustments rely on schematic perceptions, requiring minimal cognitive effort, freeing up cognitive capacity for other activity. Simulation studies demonstrate that the driving performance among novices deteriorates when cognitive demands are substantially increased, for example, by challenging memory tasks [23,24], and these effects can be greater for novice than experienced drivers [23]. Novices must focus much of their cognitive capacities on managing the vehicle and have little spare capacity for other activities. As drivers become more skillful and driving more automated, the cognitive demands decline, allowing drivers to attend to additional tasks with relative safety.

From an evolutionary biology perspective, humans are adapted with the ability to learn to react quickly (autonomously) based on subtle environmental cues. Kahneman [25] describes this as “acting fast”. Human brain activity can also be slow, and this would seem to be the state the human brain prefers most of the time. Basically, according to Kahneman, despite its amazing capacity, the human brain is lazy and resists attending to complicated subjects for prolonged periods of time, at least when there are easier and more pleasant preoccupations. Consider how difficult it is to read a research paper with the television on nearby because our attention is drawn away from the reading task, with its demand for a high degree of concentration, to watching television and its passive, pleasant, reinforcing and limited cognitive demand. Kahneman [25] describes the substantial research documenting the lazy brain phenomenon, where cognitive attention is drawn away from more challenging, difficult, complex, rote and boring activities to easier, more pleasant, brighter, dramatic and reinforcing stimuli. Similarly, beyond the early thrills from making a vehicle accelerate rapidly or turn sharply and the occasional near miss, driving is mostly a fairly boring activity, even for novices, often less interesting than other attractive options to which drivers can attend. Not surprisingly, drivers routinely listen to the radio, talk on the phone, text, groom, eat and otherwise engage in other activities that are momentarily more interesting than driving tasks [21]. While these secondary tasks may be dangerous, particularly if they take the driver’s eyes off the forward roadway, they are particularly dangerous for novices whose cognitive capacity is limited by the high demands of driving tasks.

### 2.3. Training for Licensure or Expertise?

The question, then, is how and to what extent training and supervised practice can accelerate the prolonged process required for novices to develop safe driving expertise. In North America, formal training requirements for licensure vary by country/jurisdiction, but generally, in addition to passing a written test, about 6 h of on-road training with a professional instructor are needed, probably enough to assure basic vehicle management skills [26], but not enough for most novices to develop safe driving expertise. More extensive professional driver training is required or encouraged in many other countries [5,27]. In general, the required driver education appears to do a good job of preparing novices to pass the required driving tests [28]. However, in many jurisdictions, particularly in North American, Australia and New Zealand, most of the on-road practice obtained during the learner license period is through parent-supervised practice driving. Both the required and actual amount of supervised practice driving varies considerably, but the current recommendation in the U.S. is 50 h [4], and in some jurisdictions, 100 h or more may be required, although there is no empirical base of evidence for these amounts [3]. Generally, supervised practice driving occurs almost exclusively during the learner license period, with parents seldom riding with their newly-licensed teenage drivers [29].

Regardless of the amount of training and practice, its effectiveness depends on the extent to which it prepares novices for independent driving. Accordingly, to maximize the effectiveness of training, either by professional instruction or by parent supervisors, it is necessary to consider what novices need to learn to become safe drivers and the extent to which these competencies are taught and acquired.

#### 2.3.1. Rules of the Road

Adequate knowledge of the laws that govern driving in the jurisdiction is universally required as part of the license-testing process. This knowledge can be learned by studying the rulebook and through the required classroom portion of driver education classes. Success in passing these tests is reportedly high among novices after completing driver education classes [28]. In general, this knowledge would seem to be necessary for safety, but not sufficient for the development of expertise consistent with independent driving safety.

#### 2.3.2. Vehicle Management Skills

Skillful management of the vehicle, including steering control, speed regulation, smooth acceleration and stopping, lane maintenance, backing up and turning, is understood to develop reasonably well in most novices with relatively little practice and instruction, in about 6 h according to one of the few studies on the topic [26], which is consistent with the common requirements for on-road professional instruction and the practical experience of professional drivers and supervising parents. Vehicle management skills represent explicit knowledge and ability to perform the tasks in controlled situations, but not necessarily the ability to apply this skill correctly in dynamic and complex environments. While capable vehicle management is necessary for driving safety, it is not necessarily sufficient. Indeed, an over-emphasis on vehicle management skills may suggest that safety depends on these skills rather than on attention and self-control.

#### 2.3.3. Advanced/Higher Order Driving Skills

A wide range of advanced and interrelated driving skills is required for safety. These higher order skills include the qualitative ability to perform explicit skills in dynamic and complex situations, requiring immediate, complex decision making that relies on efficient judgment about what actions to take in particular contexts [9]. Advanced skills apply the explicit knowledge gained in practice to dynamic driving situations. Examples include managing speed in traffic; judging the speed, location and intentions of other vehicles when turning, changing lanes, merging and stopping; and responding safely to the actions of other road users and other potential hazards. Other advanced driving skills include communicating with other drivers by signaling your intended actions and interpreting theirs; navigating and way finding; applying speed and maneuvering skills in traffic; attending to important elements of the roadway; managing distraction; controlling the vehicle environment; and exercising self-control. These are expert skills consistently exhibited (more or less) by experienced drivers, but less consistently among novices.

It is largely unknown how much practice and training is required for these advanced skills to develop to assure safe driving capabilities; nor is it known the extent to which they are learned as part of driver training, supervised practice or independent driving. While instructors and supervising parents can usually notice improvements in novices’ vehicle management skills as they gain experience, it is difficult to determine the extent to which advanced skills have been mastered in part because safety is the first priority of trainers’/supervisors’ who control the driving environment and conditions and influence driving behavior by being present in the vehicle, all of which to some extent can mask deficiencies in advanced driving skills. As noted, there is evidence that newly licensed teenagers make many mistakes, despite substantial training and supervised practice. Research examining young driver crash causes reported in driving records [16,30] found that driving errors contributed to many crashes (in this research, it was not possible to determine if these errors were due to a lack of skill or to inattention). In a naturalistic study of 42 novices during their first year of independent driving and their parents driving the same vehicles, Pradhan and colleagues [31] reviewed the video footage and coded the performance of drivers as they navigated the same intersections and merges (complex driving situations). Accordingly, young drivers tended to execute appropriate safety behaviors (the ones they learned in driver education, such as looking in their mirrors and signaling) initially more often than experienced drivers, but over time, the young drivers’ performance declined and gradually approached that of adults. The authors concluded that young drivers tended at first to perform the explicit skills they were taught by rote without attention to context, while experienced adult drivers were more likely to apply these skills when necessary, for example, signaling when other vehicles were present, but not otherwise. Over time with experience, novices gradually adopted the same pattern of context-related behavior.

Test track research has shown that novices are not as good as adults at certain advanced driving skills. Notably, newly licensed teenage drivers were more likely than experienced adults to run a red light when given a cell phone task as they approached an intersection [32,33]. Even after one year and an average 3000 miles of independent driving, performance on this intersection stopping behavior task did not improve and was much worse than that of experienced adult drivers [33]. While teenagers were much better at the phone task, they were not nearly as good as experienced drivers at dividing their attention such that they would look up from the dialing task in time to see the light change and bring the vehicle to a stop.

Research on driving errors among novices has translated widely into a focus on improving driving skill, ignoring for the most part a focus on the underlying reasons novices make mistakes, which is their tendency not to pay attention.

#### 2.3.4. Attention

Vehicle management skills are not helpful if the driver does not pay attention to the forward roadway so that appropriate safety maneuvers can be exercised. Both novices and adults engage in a dizzying array of tasks secondary to driving, including eating, grooming, reaching for objects and using phones and other electronic devices [20]. Even when paying attention, novices are not as good as adults at identifying potential hazards [32]. Fortunately, it may be possible to improve hazard detection skills through training [34,35], although these skills are undermined by novices’ tendencies to engage in secondary tasks that take their eyes off the forward roadway [20] because hazards cannot be avoided if not seen. It is not so much that teenage drivers engage in more secondary tasks than adults, who are also guilty of this risky behavior, but they are at greater crash risk when they engage in these tasks. This may be because they attend to these tasks for dangerously long periods of time [20], but possibly also because they are willing to engage in these tasks in more complex and potentially dangerous driving conditions than experienced adults [36]. Curiously, attention is difficult to teach during training because no responsible supervisor would allow the attention of a driver-in-training to drift. Learning to maintain attention to the driving task requires a discipline that novices must learn on their own when no supervisor is in the vehicle.

#### 2.3.5. Self-Control

The in-vehicle environment and driving context are important safety considerations, particularly for novices, but during training, these conditions are generally controlled by the trainer/supervisor (appropriately so); hence, for the most part, the novice must figure out during independent licensure how to deal with variable driving conditions and control the vehicle environment. Notably, the presence of a teenage passenger, particularly more than one, doubles the fatal crash risk for novice teenagers [37,38], but the presence of an adult passenger decreases risk among drivers of all ages [37]. Presumably, this is because adult passengers co-drive, warning the driver of possible hazards and helping to navigate (just like supervisors during training), while at least some teenage passengers engage in distracting behaviors or otherwise contribute to norms favoring risky driving. Adult passengers are likely to encourage relatively conservative and safe driving norms (tacitly, if not explicitly), while at least some teenage passengers exert risk-accepting norms. In one study, novices who reported that their friends engaged in risky driving behavior, relative to novices who reported few friends who engaged in risky driving behavior, had higher rates of Kinematic Risky Driving (KRD), in the form of elevated gravitational force events from hard stops and sharp cornering, and higher crash/near crash rates, reflective of the importance of norms on driving behavior and safety [39].

In simulation studies, novice teenage male drivers exposed to risk-accepting teenage passengers (in one study, only norms regarding risk were manipulated, and in another, confederate passengers exerted mild pressure to engage or not engage in risky driving, depending on the condition to which they were randomized) engaged in more simulated risky driving than drivers exposed to risk-averse teenage passengers, providing evidence of peer influences on risky driving [40]. In this same research, the novice teens who were sensitive to exclusion (were relatively upset at being excluded in a computer gaming task) were more sensitive to the social influences of the passengers [41]. In addition to norms, there are other challenges to self-control while driving. Notably, elevated mood, including from loud music, can affect driving performance [42], which could explain why a relatively self-controlled teenage driver who would otherwise exhibit safe driving behavior impulsively speeds or otherwise takes a big risk. If novices are going to have variable states of mind when they drive with young passengers, play loud music and engage in secondary tasks and encounter inclement weather, heavy traffic and other hazardous driving conditions, they need to be trained to avoid and manage these conditions. Hence, for training to be effective, it needs to focus on self-management skills related to managing the in-vehicle environment, maintaining attention and driving in a restrained, disciplined and safety conscious manner.

Relatedly, while the dangers of impaired driving due to drinking or drugs are part of most driver training programs and many parents admonish their teenage children not to drive impaired, it seems unlikely that novices obtain sufficient training in the practical skills required to avoid driving after drinking or drug use (DWI), riding with an intoxicated driver (RWI) or managing passengers who are impaired. Despite declines in recent years, substance use, DWI and RWI are not uncommon, at least among U.S. teenagers [43].

#### 2.3.6. Individual Variability

It should be noted that there is wide individual variability in risk among novice teenage drivers, due presumably to differences in maturity, norms, social influences and so on. While inexperience is the most important factor in crash risk, young age is also an important factor [2]. However, there is wide individual variability among young and inexperienced drivers. Notably, many novices experience no crashes, seldom engage in risky driving and exercise mature driving judgment. Guo et al. [44] demonstrated in a naturalistic driving study that about 1/3 of the 42 novice participants had no or very few Crashes or Near Crashes (CNC) during the first 18 months of licensure. Another 1/3 had many CNC, and their rate did not decline over the 18-month study period. Another 1/3 had a high CNC rate for about six months, which then declined rapidly to approximate that of the safest third. The authors concluded that some novices were more prone to risk than others, while other novices required the feedback from experience to learn how to improve driving performance. In that same naturalistic study, KRD rates were also variable both within and between drivers [45]. About half the sample exhibited a trajectory over the 18-month study period of relatively lower KRD rates, while the other half exhibited relatively higher KRD rates (the correlation between average KRD rate and CNC rate was *r* = 0.60). The possible reasons for the relatively high variability in KRD over time in both groups (higher in the high rate group) include driver experimentation, driver mood, driving context, passenger presence and individual characteristics of drivers. One other finding in that study was that speeding, defined as the rate of driving 10 or more miles or kilometers over the speed limit, also varied considerably among young drivers [46]. Various implications can be drawn from these findings that risk varies among young drivers. It seems that some novices are safer than others, while some learn from substantial experience, and still others seem not to learn as well from experience or may learn, but prefer to drive in a more risky manner despite the consequences. Further, the same teen may be relatively safe under some conditions, but engage in risky driving behaviors in certain situations, for example in the presence of certain passengers, at night or on particular roadways.

The characteristics of the individual variability in driving performance have been difficult to fully elucidate, despite substantial research. Incomplete brain development has frequently been mentioned in this regard [47]. The best evidence for an effect of incomplete brain development is the high rate of crashes among adolescents with ADHD [48], whose brains tend to lag a year or so behind age peers on some dimensions. Of course, there is wide variability in brain development among adolescents generally [49]. While the brain does not fully develop until at least the early 20s, by age 16, the brain is substantially developed such that most adolescents are highly capable of self-controlled behavior. However, there is little question that relative to adults, adolescents are more willing to accept risk, have less cognitive control and are more sensitive to social reward and peer influence [49,50]. Hence, while adolescent brain development probably plays a role in driving risk, the extent and nature are not established.

Many other measures of individual variability have been studied. A number of studies has shown that perceived norms regarding safe driving behavior are associated with self-reported driving outcomes [51], consistent with the literature on reasoned action variables [51,52]. One study demonstrated an association between stress responsivity and crash risk [53]. Theoretically, adolescents who do not respond normally to stress may be inclined to engage in behavior that would seem risky to others, but not themselves. Sensation seeking has sometimes been associated with increased risk [54], but not consistently. In general, with rare exception [54], personality measures have not been shown to explain very much of the variance, although young drivers scoring higher on conscientiousness may be at somewhat lower risk [55]. Unfortunately, despite substantial research on measures of individual variability, the amount of the variance that can be explained by any one or combinations of co-variates is modest, and it is not altogether clear why some novices are so much more risky than others.

### 2.4. Some Limits of Training Potential

It is evident that the challenges involved in training young novices to drive safely are substantial. Clearly, both training and supervised practice can facilitate improvements in basic driving and vehicle handling skills and may be adequate to fulfill these learning needs. However, considerably more needs to be learned about advanced skills, attention maintenance and self-management. Despite numerous evaluations, there is little evidence that driver education as it is currently delivered or requiring many hours of supervised practice driving provides the skills necessary to reduce crash outcomes [27,56]. While extending the practice driving period and requiring substantially more supervised practice driving prior to licensure are logical policy objectives, they should be considered only part of the solution to the young driver problem.

#### 2.4.1. Extending the Learner Period

While driver education appears to do a good job of teaching the rules of the road and explicit knowledge and skills related to vehicle management, the limited number of hours behind the wheel with professional trainers could not be expected to foster expertise consistent with safety. The amount and nature of training and supervised practice driving that most novices need to become nominal experts is not known. Notably, despite enthusiastic adoption in many jurisdictions of longer learner periods and increased supervised practice driving requirements, there is no convincing evidence to date that the amount of supervised practice driving is associated with safer independent driving outcomes [3]. The great advantages of a long learner period include the potential for increased amounts of supervised practice driving over a longer period of time, increased exposure to novel road conditions (fostering associative learning opportunities), opportunities for extensive corrective feedback and the opportunity for supervisors to establish their expectations for safe independent driving behavior. Therefore, more training over the longest period possible is probably best, but practice driving alone may not be sufficient. Indeed, Foss [3] has argued that requirements for greater amounts of practice driving may erroneously suggest that increasing independent driving safety among newly licensed novices is simply a matter of increasing the amount of pre-licensure supervised practice, which is unlikely to be the case [9–13]. For practice driving to be most effective, it might need to expose novices to more complex driving conditions and focus on higher order instruction, self-control and safe driving attitudes, thereby improving the translation of learning to independent driving.

##### Co-Driving

Because safety is paramount, driving instructors and supervising parents commonly co-drive to prevent unsafe driving. Co-driving includes navigation (“turn left at the next corner”; “get into the left lane”), guiding behavior (“stay in your lane”), providing warnings (“look out for that car”), anticipating maneuvers (“slow down now before making a turn”), pointing out potential hazards (“a pedestrian might emerge from that hidden crosswalk”), restricting secondary tasks (“do not answer the phone while you are driving”), maintaining driver attention (“look forward and then glance briefly in your mirrors and back to the forward roadway”) and controlling the vehicle environment (“turn down the music”; “put down your phone”). In a naturalistic driving study of supervised practice driving, analyses of audio-taped conversations indicated that much of the driving-related communication by parents represented these forms of co-driving [57]. Ehsani et al. [58] reported similar findings with a few cases where supervising parents attempted to generalize learning from a particular situation (“slowdown in this neighborhood”) to a general class of behaviors (“in neighborhoods, you have to drive slowly because children and pets can run out from between the parked cars”) or their expectations for independent driving (“when you drive on your own, I do not want you ever to dial your phone or text”). While co-driving keeps novices safe (the crash rate during supervised practice is low), the novice does not gain experience making independent decisions about behaviors like these until beginning to drive independently. Ideally, as novice driving skills improve, supervising adults would expose them to a wider range of and more complex driving conditions, simulating an environment that is as close as possible to independent driving.

While supervisors can emphasize how important it is for the young driver to pay attention to the driving task, not engage in distracting secondary tasks, control the vehicle environment and remain self-controlled, in practice, these are things the novices will mostly need to work out for themselves during the early months of independent licensure when the novice is confronted with choices about allocating attention to demands from driving, passengers, media and other tasks and challenges to self-control from mood, impairment and social influences.

### 2.5. Improving Training and Supervision Effectiveness

Could new approaches to training and supervised practice improve judgment, attention, automaticity and ultimately improved performance and lower independent driving crash rates? It has long been lamented that most professional driver education programs are of short duration and focus primarily on basic vehicle management skills, leaving little time for higher order and advanced skills consistent with independent driving safety. Indeed, we contend that there are inherent limits on the current model for training, not the least of which is that novices will always need to learn how to do things on their own without the instructor in the vehicle. Researchers and driving professionals in many countries have recognized the limits of the current driver training paradigm and are exploring new approaches that stretch the boundaries of current practice. While most of these approaches focus on advanced vehicle management skills and some on allocating attention (e.g., hazard perception, situational awareness), less of this research has focused on the translation of learning from the learner period to independent driving.

#### 2.5.1. Training Innovations

A recent review of the literature on the effects of driver training on crash outcomes by Peck [59] that included studies from North America, the U.K., Australia, New Zealand and Scandinavia noted the lack of well-controlled studies and conflicting findings. Peck concluded optimistically that conventional driver education training might possibly reduce per licensed driver crash rates by 0%–5% over the first 6–12 months of driving and recommended greater emphasis on attitude change and risk taking. Two isolated European evaluations have reported encouraging program effects [60,61]. Those exposed to a new driver training program in Denmark introduced in 1986 had better crash records than those exposed to the previous, standard training program, while in Austria better crash outcomes were reported by drivers who completed the standard program compared with drivers who opted out of driver education. However, participants self-selected into these programs, so selection bias may have been a factor.

Tronsmoen [62] examined the effect of different types of driver training (formal versus lay instruction) on a range of self-reported assessments of driving ability and safety consciousness Receiving more formal instruction was positively associated with safety attitudes, but was generally negatively associated with self-assessed skill and ability to drive safely. In contrast, participants receiving more lay instruction were more likely to rate their skills more highly and report more lax attitudes towards risky driving. Across both categories of instructions, the associations were modest.

Beanland et al. [27] reviewed the literature on the effects on both pre- and post-licensure driver training, with a particular focus on European efforts. This report also lamented the weak, confounded, non-randomized research designs and low power for detecting differences and concluded that the literature provides little evidence that driver training programs improve safety outcomes. They found no evidence that post-license training programs improved safety outcomes, but noted positive effects on procedural and hazard detection skills consistent with improved safety. Similarly, a review of the literature on European post-licensure training programs noted the lack of well-controlled evaluations, but concluded that their emphasis on self-assessment, anticipation of risk and driving discipline offers promise [63]. Most innovative training efforts have focused on improving advanced vehicle management, sometimes after licensure, such as procedural training and defensive driver courses. Post-license procedural training, sometimes as long as a week but often a single session, has mostly focused on advanced vehicle management skills such as skid training. One aspect of the popularity of these programs is that young drivers can drive fast and recklessly (usually on a track or field) in a relatively safe environment, learning about how to deal with eminent danger. To date, the evidence of safety effects is scant and inconsistent, and there is a growing concern that such training my increase confidence in one’s ability to maneuver to avoid a crash rather than avoid such events and possibly increase rather than decrease risk taking.

A two-phased approached is offered in Finland and Austria where learners initially get basic driver training and later advanced driver training. A recent evaluation of this two-phase model in Finland and Austria based on post-program surveys found no reported effects on self-assessed safe driving benefits, but the Finnish sample reported benefiting from the economical driving training component of the program [64]. Furthermore, one relatively comprehensive program that included one intensive week of higher-order training that included hazard perception and situation awareness training was evaluated in comparison to procedural training, with reported improvements in some skills and risky driving attitudes [65]. In general, most evaluations have been of short duration, focused on intermediate learning outcomes and not safety outcomes, and did not have strong research designs. Beanland and colleagues (p. 132, [27]) concluded that there is modest evidence for improvements in vehicle management skills, but not much for safety outcomes: “ … there is limited evidence regarding the efficacy of post-licensure training for improving on-road safety and reducing crash risk”. Nonetheless, some of these efforts, however poorly evaluated, for better or worse, are gradually finding their way into practice. For example, hazard perception skills are now a part of driver training and testing in England and Australia, and possibly elsewhere.

An alternative approach has been developed that focuses on more general (than driving) personal, self-management skills. An evaluation of such a program, using a prospective cohort design, was conducted with over 20,000 Australian youth who self-selected to participate in one of two programs, each of which included standard driver education activities plus an additional one-day workshop [66]. In one program, the workshop focused on driving risk and road safety and included police, driving instructors and others. The workshop for the other program focused not only on driving risks, but also on personal skills related to risk taking, resilience and empowerment; additional follow-on community activities varied by region. The evaluation indicated reductions (compared with those who took standard training and neither of the workshop programs) in crash rates for the resilience/empowerment group, one of few such reports. The program used driving as the context for self-skills, norms and attitudes rather than skill development for safe driving. Unfortunately, no intermediate outcomes were reported, so it is unclear what those in the resilience/empowerment program learned that might have contributed to safer outcomes. The non-randomized design makes generalization uncertain, and more research on such approaches is needed.

The Goals for Driver Education (GDE) matrix has been influential in guiding the development of driver training programs in Europe, where the model originated [67]. In brief, GDE proposes a hierarchical model for driver training that begins with the basic knowledge and vehicle handling skills needed for driving (Level 1) and progresses to the translation of those skills to a dynamic roadway context, where drivers are able to adapt to a variety of situations (Level 2). The third level of the GDE model posits that drivers consider the factors involved at a journey level, such as the purpose of the trip, the prevailing driving conditions and the social context and passengers who may be accompanying them. The fourth level is related to drivers’ life goals and purpose. In this sense, the GDE framework is unique in recognizing the importance of motivation as an essential training element. However, the role of life purpose and motivation are not likely to change drastically in the first six months of driving, when crash risk declines most dramatically. Moreover, it has been difficult to incorporate these lofty concerns into effective practice. Despite relatively widespread adoption, at least conceptually, little evidence for the effectiveness of GDE-based training exists. A rare evaluation of a one-day training program based on the GDE framework in Spain found that participants assigned to the intervention reported significantly higher skills for safe driving following a nine-month follow-up period. However, the magnitude of the effect was small, and there was no significant difference between the groups in four other self-reported dimensions of safe driving that were assessed at follow-up [68].

Hazard skill training is an innovation that is rapidly being adopted in practice. Computerized hazard skill training is a part of license testing in England and Australia, and possibly elsewhere, although more evaluation is needed. A thoughtful series of simulation studies demonstrated that novices are not as good as experienced adults at identifying and responding to potential road hazards, improved with training in a simulator and that the training transferred to performance on a road test [35]. Subsequently, the training has been expanded and adapted for use on desktop computers, allowing broader application. A recent evaluation reported that an evaluation of novices exposed and not exposed to a brief training protocol showed differences in crashes the first year after licensure [69]. Should this surprising preliminary finding hold up, it would suggest the potential benefits of integrating hazard skill training into driver education and testing. However, it remains unclear if the training influences learners’ attention to the forward roadway or mainly to the ability to recognize hazards when seen. The growing enthusiasm for hazard skill training is warranted, given the potential to extend this training widely through computer and simulation training, but is tempered by the recognition that hazards can only be identified and mitigated when the driver attends to the forward roadway and driving task, resisting distractions from secondary tasks, passengers and other threats to attention and self-control.

A summary of the evidence, potential and limitations of existing teen driving safety programs is presented in Table 1. In general, the evaluations (mostly uncontrolled evaluation designs) of most innovative training approaches have been equivocal, with some positive effects reported for knowledge and skill outcomes, but it is not clear if any of these ideas can improve safety; and some may even have negative effects on safety. The broad utility of one or a combination of these novel approaches may eventually prove generally useful. It is notable that improvements in important skills can be increased through focused training. However, it is unclear how training can provide safety effects if it is not able to change the eventual independent driving behavior of novice young drivers with respect to attention, secondary task engagement and self-control.

#### 2.5.2. Supervised Practice

Supervised practice driving suffers from some of the same limitations as driver training. Generally, the more practice over the longest possible period in the widest possible driving conditions would seem optimal, but as noted, it is not simply a matter of the amount of practice, but the extent of deliberative practice that matters [3,13]. Moreover, the parent or other adult supervisor is in the car co-driving and otherwise controlling many aspects of the driving situation and context. After all, teenagers do not always behave the same way on their own as they do when a parent or other adult is present. Therefore, how can parents and supervisors effectively extend their expectations and influence into the vehicle vicariously? Longer periods and greater amounts of practice driving provide more opportunity for parents to influence their teenage children; but it is not clear that they make the best use of these, and it seems they make little effort in this regard. As noted, most parent communications in the vehicle focus on instructions, directions and other co-driving behavior, with little focus on higher order skills and almost nothing on the parents’ expectations, requirements and consequences for violating these expectations when the teen drives independently. There is evidence from one study that parent or lay supervised driving may be as good as professional training [70]. One study demonstrated that parent adoption of a driving plan was associated with increased parent-supervised practice driving [71]. Supervisors could use the driving context to provide higher order instruction, helping the novice generalize specific driving situations to others like they may eventually encounter, and establish in the mind of the novice the following: norms for safe driving behavior; the priority of attending exclusively to the driving task and refraining from engaging in distracting secondary tasks; controlling the vehicle environment and themselves when angry, upset or excited. However, it appears that parents provide little higher order instruction [57,72], and professional supervisors may not be appreciably better [73].

#### 2.5.3. Parental Management of Independent Driving

GDLS restrictions on independent driving during the provisional stage provide the opportunity for parents to influence independent driving. Parents are mainly responsible for assuring that their teenagers abide by the GDLS provisions [74]. Some efforts have been made to encourage parents to go beyond GDLS limits, restricting teenage driving earlier in the evening, under inclement weather conditions, with no or only one passenger, and so on. Randomized trials have demonstrated that it is possible to deliver interventions directed at parents in a variety of settings, including driver education, with significant effects on parental management, including the adoption of a parent-teen driving agreement, the establishment of more strict limits on independent driving and lower rates of risky driving and crashes [75–77]. Qualitative research of teen drivers’ perceptions of their parents [78] suggests parental expectations play an important role in promoting safe driving among novice teens. Parental management of novice teenage driving can now be facilitated with technology, including a variety of devices with accelerometers that measure elevated g-force events, a reliable measure of risky driving [79]. However, for these monitoring devices to be effective, parents need to establish expectations and consequences for risky driving behavior [80].

#### 2.5.4. Testing for Licensure

A certain way to reduce crash rates is to make licensure more difficult because it delays licensure and reduces exposure. In countries with very difficult tests, for example Japan and England, licensure typically occurs at older ages than in the U.S., where these tests are generally not difficult. A recent NHTSA report documented license testing in the U.S. and found that testing quality and difficulty varied little from state to state, and no evidence was found that the states with more difficult tests had lower crash rates [81]. While in the U.S. there has been no discernable, recent change in testing, Australia, Canada and New Zealand, for example, have upgraded testing requirements in conjunction with GDLS upgrades. Australia and England have imposed a computerized hazard skills test as part of licensing, but no published evaluations are available. While there is potential for testing to improve training as driving schools and families adapt to more strict requirements, this is not currently the case in the U.S., and it is unknown the extent to which changes in testing requirements translate into improved training.

#### 2.5.5. Technology

The rapid growth of technology has changed many aspects of modern life, with the rapid onset of automated vehicles a case in point. Clearly, the solid advances in vehicle safety in the form of safety belts, air bags and crashworthiness, as well as safer road designs make driving safer for all drivers. Technology would seem to have potential to improve the learning to drive process. Notably, evaluations of DriveCam, a device with cameras mounted near the rear view mirror that records the video of the seconds around elevated gravitational force events, have demonstrated significant reduction in kinematic events consistent with less risky driving [82]. However, recruitment of families and participation on the part of parents has been disappointing. Moreover, parent participation would seem to be necessary because the behavior of novice teenagers does not appear to change simply by providing a warning that they exceeded a gravitational force limit. In one study, young drivers with DriveCam devices installed in their vehicles drove with the device in “stealth” mode for one month, providing baseline values. Then, the study participants were randomized to one of two groups: (1) feedback to the driver only; or (2) feedback to the driver and to the drivers’ parents of two treatment groups. When the feedback (in the form of a blinking light) was activated, the g-force rates of the second group declined immediately and significantly, while the rates for the feedback to driver only group did not decline [82]. The authors concluded that only contingent feedback affected behavior. There are many related and unique technologies available or in development, including devices that prevent cell phone operation while driving, devices for measuring and providing feedback regarding kinematic events, etc. Technology is a likely partner in future efforts to improve driver learning and behavior, but it seems that in most cases, technology needs willing learners who are motivated to drive safely.

### 2.6. Discussion

#### 2.6.1. Future Research and Measurement

The research base on training young drivers is limited by the few studies and small budgets available for this research. Promising approaches have not been well evaluated, and many of the programs, at least as evaluated, did not have the benefit of numerous iterations and repeated trials that would help to identify the key elements of the approach. While some studies focused on intermediate outcomes, mostly vehicle management outcomes, more measurement of intermediate outcomes, both self-reported and objectively assessed, is needed to determine if the objectives of the program were achieved. Most importantly, it may be unrealistic to expect programs to achieve measurable safety outcomes, such as crash rates, given the small sizes of most evaluable programs and the relative rarity of crashes. While there are too few measures of intermediate outcomes linked to crash outcomes, the technology is advancing rapidly and it should be possible to use accelerometers increasingly to assess kinematic risky driving and in-vehicle cameras to assess secondary tasks. Despite substantial research on the topic, we do not have reliable and convincing psycho-social models of risk that would enable the assessment of intermediate outcomes and facilitate the development of appropriate interventions.

#### 2.6.2. Training and Supervision as Part of Comprehensive Risk Management

Where adopted, GDLS policies have been demonstrated to effectively reduce crash risk, but these policies are in place only in certain countries with relatively young age at licensure, and where adopted, they are less strict than recommended. In any case, these policies depend greatly on parental management and also provide parents with a great opportunity to establish safe driving expectations during the prolonged practice driving period. It is clear that current practices in driving education and parent supervised practice driving, while effective in achieving the proximal goals of training novices to manage the vehicle reasonably well and pass the driving test, need to be augmented and reoriented toward the transition to independent driving and the major causes of novice driver crashes: distraction, poor judgment, lack of self-control and inadequate motivation for safe driving. The task for training and supervision is to foster improvements in these complex skills that translate into safety benefits during the early months of independent driving. The next set of advances in teen driver risk reduction may be informed by the ongoing research on innovative training and supervised practice driving methods that seek to foster better transfer of learning from the learner stage to safer driving behavior during independent driving.

## Figures and Tables

**Table 1:** Novice teen driving safety programs: do they improve safety? Evidence, potential and limitations.

Program	Goals	Evidence of Safety Benefit	Potential	Limitations
Graduated Driver Licensing Systems (GDLS)	Limit exposure	Substantial evidence	Population impact, strict limits are better; enforcement is lax	Policy balance between safety and mobility; limited to a few countries
Driver education	Vehicle management; prepare for license tests	Many evaluations show no safety benefits	Mandatory in some jurisdictions, popular program; potential innovations identified	Few practice hours; not linked to GDLS or parent supervision and management
Practice driving	Provide experience; improve skills	Few evaluations	Extensive practice; consistent with effective parenting practices	Little evidence of higher order instruction or emphasis on independent driving norms, expectations
Parental management	Limit exposure; set expectations	Benefits shown in few studies; low participation	Parents can, but do not set limits; low parent participation; could be linked to driver education and GDLS	Low parent enthusiasm and participation

## Abbreviations

GDLS: Graduated Driver Licensing
GDLS: Graduated Driver Licensing Systems
GDE: Goals for Driver Education
KRD: Kinematic Risky Driving
CNC: Crashes or Near Crashes
DWI: Driving While Intoxicated
RWI: Riding With an Impaired driver
NHTSA: National Highway Traffic Safety Administration
